# Chronic Granulomatous Interstitial Nephritis Presenting Five Years After Intravesical Bacillus Calmette-Guérin Therapy: A Case Report and Literature Review

**DOI:** 10.7759/cureus.106712

**Published:** 2026-04-09

**Authors:** Mehdi El Mansouri, Hanane Benali, Ismail Ait Elkihal, Nabil Hamouche, Mariam Chettati, Wafaa Fadili, Inass Laouad

**Affiliations:** 1 Nephrology, Mohammed VI University Center Hospital, Marrakech, MAR; 2 Nephrology, Faculty of Medicine and Pharmacy, Cadi Ayyad University, Marrakech, MAR

**Keywords:** bacillus calmette-guerin, bcg renal complications, drug-induced acute renal failure, granulomatous interstitial nephritis, intravesical bcg therapy, late bcg toxicity, non-caseating granuloma, non-muscle invasive bladder cancer, systemic bcg reaction

## Abstract

Intravesical administration of Bacillus Calmette-Guérin (BCG) has been the standard treatment for non-muscle invasive bladder cancer (NMIBC).

Its efficacy in preventing recurrence and tumor progression has been well established; however, rare immune-mediated complications, particularly parenchymal renal involvement, pose significant diagnostic challenges. This report describes an exceptional case: that of a 73-year-old man who developed renal failure five years after his last BCG instillation. Histopathological examination revealed granulomatous interstitial nephritis. This case is remarkable for its exceptionally long and unusual latency period, which generally manifests within days to months in other patients.

After initiating corticosteroid therapy, the patient experienced partial renal recovery and stabilization of renal function during follow-up, highlighting the potential role of immunological mechanisms in delayed renal injury.

This study underscores the need for long-term renal monitoring in patients treated with BCG immunotherapy.

## Introduction

Bacillus Calmette-Guérin (BCG), a live attenuated strain of *Mycobacterium bovis*, constitutes the standard of care for non-muscle invasive bladder cancer (NMIBC).

First introduced in 1976 by Morales et al. [1] and FDA-approved in 1990, intravesical BCG therapy relies on a complex immunological mechanism: the bacilli adhere to the bladder urothelium, triggering a robust local immune response involving macrophages, natural killer (NK) cells, and CD4+ and CD8+ T lymphocytes. This process induces a predominantly Th1 microenvironment that promotes the destruction of tumor cells [2]. 

While this therapy is widely utilized, it is not without side effects. The most frequent complications are generally minor and limited to bladder irritation, low-grade fever, or myalgia. Local granulomatous involvement (such as prostatitis and epididymitis), as well as systemic complications--particularly pulmonary or hepatic--remain exceptional [3, 4]. Renal involvement is equally rare [5, 6]. Nevertheless, several exceptional cases of interstitial nephritis and glomerulonephritis, with or without associated granulomatosis, have been described in the literature [5-8].

We report herein a case of granulomatous interstitial nephritis occurring late after intravesical BCG instillations in a patient being monitored for low-grade NMIBC.

## Case presentation

Clinical history and initial evaluation

A 73-year-old male of North African descent with a 10 pack-year smoking history was referred to the nephrology department in November 2022 for the evaluation of severe renal impairment, which was incidentally discovered during routine laboratory testing (estimated glomerular filtration rate (eGFR) of 11.7 mL/min/1.73 m²).

His medical history was significant for low-grade, stage pTa non-muscle-invasive urothelial carcinoma diagnosed in 2016, which was managed via transurethral resection followed by standard intravesical BCG immunotherapy. He had no prior history of chronic kidney disease, recurrent urinary tract infections, or recent exposure to nephrotoxic agents.

At presentation, the patient reported a several-month history of progressive asthenia. Physical examination revealed a hemodynamically stable patient (blood pressure: 137/69 mmHg) with no evidence of systemic inflammation, skin rash, or lymphadenopathy. Respiratory and cardiovascular examinations were unremarkable. Initial laboratory findings disclosed a serum creatinine level of 404 μmol/L, corresponding to a eGFR of 11.7 mL/min/1.73 m². Urinalysis demonstrated hematuria (++) and trace leukocytes, whereas urine cultures were negative for both commensal bacteria and acid-fast bacilli.

Diagnostic workup

To exclude alternative etiologies, a comprehensive evaluation was conducted. Immunological assays (antinuclear antibodies (ANA), anti-double-stranded DNA (anti-dsDNA), ANCA, and anti-GBM antibodies) were all negative. Serum complement levels (C3/C4), glycated hemoglobin, and viral serologies (hepatitis B and C) were within normal limits. Screening for multiple myeloma and systemic sarcoidosis was also negative, supported by normal ACE levels and an unremarkable chest computed tomography (CT) scan, which was performed to definitively rule out hilar or pulmonary involvement. Proteinuria was quantified at 0.4 g/24 h.

**Figure 1:**
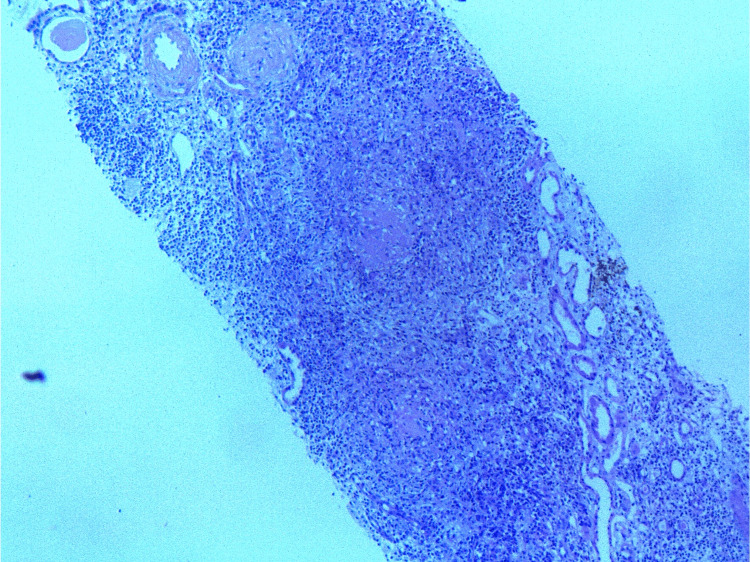
Medium-power view of a renal biopsy core (Masson's trichrome stain). Renal biopsy core at medium magnification (Masson's trichrome stain). The section reveals 11 glomeruli, including 4 with global sclerosis, and atrophic glomeruli with prominent mesangial sclerosis. The interstitium is widened by a diffuse inflammatory infiltrate featuring non-caseating epithelioid and giant cell granulomas. Thickened arterioles with reduced lumens and approximately 45% tubular atrophy are also observed.

**Figure 2:**
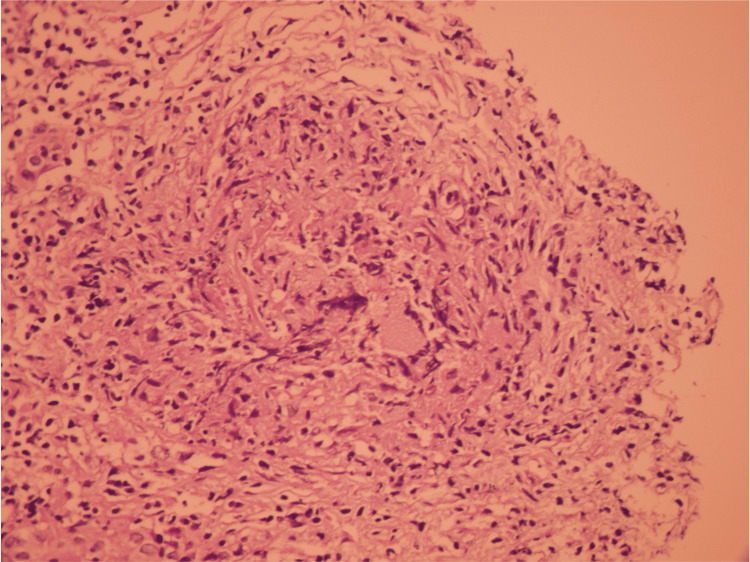
High-power view demonstrating a granuloma predominantly composed of epithelioid histiocytes admixed with lymphocytes.

An ultrasound-guided renal biopsy was performed to investigate the unexplained renal impairment. Histopathological examination revealed glomerulosclerosis alongside an interstitial infiltrate consisting of lymphocytes, eosinophils, and histiocytes, organizing into non-caseating epithelioid and giant-cell granulomas (Figures 1-2). Background features included chronic tubular atrophy and thickened arterioles with luminal narrowing, notably without evidence of tubulitis. Immunofluorescence staining was negative. Furthermore, no acid-fast bacilli (AFB) were detected, and a polymerase chain reaction (PCR) assay (Xpert MTB/RIF) performed on the renal biopsy tissue was negative.

Management and outcome

Despite a five-year latency and the presence of chronic histological lesions, corticosteroid therapy was initiated to target the active inflammatory component. The patient received methylprednisolone, followed by oral prednisone (1 mg/kg/day, rapidly tapered over eight weeks). The eGFR improved from 11.7 to 24 mL/min/1.73 m² after two months of follow-up.

Following this partial recovery, renal function stabilized. Over an 18-month follow-up period, the eGFR remained stable between 19 and 20 mL/min/1.73 m². Of note, there was no evidence of systemic BCG infection, and repeat cystoscopy revealed no recurrence of NMIBC.

## Discussion

Intravesical instillation of BCG, utilizing a live attenuated strain of *Mycobacterium bovis*, is a highly effective and widely utilized therapeutic modality for in situ and low-grade NMIBC. Although its precise mechanism of action remains incompletely elucidated, current evidence suggests a robust local immune response mediated by CD4+ and CD8+ T lymphocytes, NK cells, granulocytes, macrophages, and dendritic cells. Furthermore, neoplastic urothelial cells actively participate in this cascade by adhering to and internalizing BCG, thereby stimulating the production of cytokines and chemokines, as well as presenting BCG and/or tumor-associated antigens to immune system cells [2].

The tolerability profile of intravesical BCG therapy is generally favorable, with minor and transient adverse effects occurring in 10-50% of patients. These manifestations typically present as flu-like symptoms, general malaise, and localized bladder irritation resulting in dysuria [9]. Severe local or systemic complications, such as granulomatous lesions, abscesses, and pyelonephritis, are rare [10, 11].

More recently, however, a growing body of literature has highlighted immune-mediated complications, including hypersensitivity reactions, Henoch-Schönlein purpura, as well as renal glomerular and tubulointerstitial injuries [6, 12]. Table 1 outlines the spectrum of glomerular and tubulointerstitial lesions reported following intravesical BCG instillations; the data are summarized below.

**Table 1:** Clinical, histological, and therapeutic characteristics of reported cases of post-BCG renal toxicity. Abbreviations: I, isoniazid; R, rifampin; E, ethambutol; Pred, prednisone / prednisolone; MP, methylprednisolone; IF, immunofluorescence; CKD, chronic kidney disease; eGFR, estimated glomerular filtration rate.

Author et al., year	Age/ sex	Clinical presentation	Initial eGFR (mL/min/1.73m²)	BCG instillations (n)	Delay	Renal histology	Granuloma	Extra-renal involvement	Treatment	Recovery	Final eGFR (mL/min/1.73m²)
Modesto et al., 1991 [6]	70 / M	Altered general condition, elevated serum creatinine	20	11	6 months	Interstitial epithelioid granulomas, IF negative	Yes	Liver epithelioid granulomas, disseminated foci of chorioretinitis	I + Pred	Partial	37
Modesto et al., 1991 [6]	70 / M	Acute kidney injury, weight loss, fever	9	18	7 months	Interstitial nephritis with mesangial IgM and C3 deposits	No	Liver and bone marrow non-caseating epithelioid granulomas	I + E + Pred	None (Died)	-
Modesto et al., 1991 [6]	48 / M	Fever, visible hematuria, edema, dyspnea	64	9	Immediate	Diffuse mesangial proliferation with subendothelial deposits of IgG + C3, moderate interstitial fibrosis	No	Liver and spleen minor uptake on scintigraphy	I + R	Complete	Normal
Fry et al., 2005 [13]	72 / M	Progressive asthenia, anorexia, acute kidney injury	7	8	Unspecified	Acute tubulointerstitial nephritis, mesangial proliferation with focal segmental changes, IF negative	No	Liver function test abnormalities	I + R + Pred	Partial	34
Kennedy et al., 2006 [7]	72 / F	Acute kidney injury, fever	19	5	7 days	Diffuse interstitial nephritis, tubular degeneration, non-necrotizing granulomas, IF non-specific (IgM + C3)	Yes	None	Pred	Complete	40
Jose Manzanera Escribano et al., 2007 [5]	76 / M	Acute kidney injury	7	10	1 month	Diffuse and severe interstitial nephritis	No	None	MP + Pred	Partial	19
Singh et al., 2007 [14]	54 / M	Edema, nephrotic syndrome	Normal	12	Immediate	Membranous glomerulonephritis (IgG, IgM, C3 positive)	No	None	Pred	Complete	Normal
Al-Qaoud et al., 2015 [8]	74 / M	Visible hematuria	Not reported	9	2 months	Chronic granulomatous interstitial nephritis	Yes	None	None	Complete	Not reported
Mohammed et al., 2017 [15]	73 / F	Advanced CKD (Stage 5)	10	16	6 weeks	Interstitial nephritis with granuloma, acute and chronic tubular damage, IF negative	Yes	None	Pred	Partial	17
Tamzali et al., 2021 [16]	79 / M	Altered general condition, fever	12	2	1 day	Not performed (Presumed BCG-associated nephritis)	-	None	I + R + E	Partial	20
Present case	73 / M	Progressive asthenia, acute kidney injury	11	18	5 years	Glomerulosclerosis, diffuse interstitial nephritis with non-necrotizing granulomas, chronic tubular damage, IF negative	Yes	None	MP + Pred	Partial	24

Analysis of reported cases in the literature (Table 1) highlights the specific clinical and prognostic profile of post-BCG nephrotoxicity, while also emphasizing the unique nature of the case presented. Classically, renal complications induced by intravesical BCG instillations--whether glomerular or tubulointerstitial--manifest acutely or subacutely. Indeed, the data collected indicate a time to onset ranging from a few days to seven months after treatment [5-8, 13-16]. Consequently, the renal failure observed five years after the last instillation represents an extremely long and unusual time to onset.

From a histological and pathophysiological standpoint, the literature confirms the predominance of acute or chronic interstitial nephritis, frequently associated with non-caseating epithelioid granulomas [5-8]. The absence of viable bacilli within the lesions, combined with the frequent absence of concomitant extrarenal involvement, strongly supports the hypothesis of a delayed hypersensitivity reaction (type IV cellular immunity) rather than that of an active mycobacterial infection [5, 7].

Analysis of clinical outcomes reveals an often severe renal functional prognosis. Although corticosteroid therapy effectively mitigates the active inflammatory component, renal function recovery remains predominantly partial [5-8, 13-16]. This unfavorable clinical course may be attributable to the chronic renal damage sustained during the prolonged asymptomatic phase.

Furthermore, from a pathophysiological perspective, it is imperative to consider the potential role of pre-existing or acquired vesicoureteral reflux (VUR) in these patients. The presence of such reflux may constitute a major anatomical risk factor, facilitating the retrograde ascent of the therapeutic agent to the upper urinary tract during intravesical instillations, thereby precipitating the onset of BCG-induced inflammatory lesions.

These clinical observations strongly underscore the necessity of re-evaluating the chronological paradigm of post-BCG follow-up. We advocate for the implementation of long-term monitoring of the eGFR, potentially coupled with screening for urological abnormalities, to detect these immune-mediated lesions prior to the development of irreversible renal sequelae.

## Conclusions

Our case illustrates that intravesical BCG treatment can trigger severe granulomatous interstitial nephritis after an exceptionally long latency period of five years. Such an event challenges the currently established standard monitoring protocols for patients receiving this treatment. It underscores the importance of prolonged renal monitoring, including periodic assessments of the eGFR for several years after treatment.

Furthermore, early renal biopsy in cases of unexplained decline in renal function facilitates the rapid identification of the underlying renal pathology and early therapeutic intervention, thus preventing irreversible structural damage. Finally, although intravesical BCG remains a cornerstone of the oncological management of NMIBC, a thorough understanding of its late systemic toxicities is essential for optimal multidisciplinary clinical management.
